# Our Present Status

**Published:** 1894-12

**Authors:** T. B. Welch

**Affiliations:** Vineland, N. J.


ARTICLE II.

### OUR PRESENT STATUS.

T. B. WELCH, M. D., VINELAND, N. J.

In the present status of the dental profession we have much to be proud of, some things to regret, and many things to hope for.

We have certainly made great strides in general intelligence, specific knowledge and scientific chemical research, and in skill, material and appliances.

The profession of law has made little progress for a hundred years; the preachers are not much better than those of fifty years ago, and have physicians made improvements commensurate with their age? Dentistry, as a profession, is hardly fifty years old, yet its increase, in numbers, skill and dignity is phenomenal. Within thirty years its stately steps, its honorable standing, and its notable distinction gives us respectability everywhere. In its young energy and laudable pride it is almost ready to lock arms with the aristocracy of "the leading professionals." Really, if these learned gentlemen are less empirical, and are not more specific in their prescriptions and more broadly scientific and exact in knowledge, we shall even outstrip them in the race for the world's honors and confidence.

It is true, these medical men have the advantage of us, for "dead men tell no tales;" and, even with patients that live, the evidences of fraud and incompetency are not easily proved. In view of the blunders and mal-practice of the many, a venerable president of the London Medico-Chirugical Society once declared in his annual address, "I verily believe if all the doctors and surgeons, and mid-wives and druggists, and drugs, were hurled from the face of the earth, there would be less sickness and less mortality among the people. But if we dentists show incompetence our patients live to curse us. Our poor work fairly grins at us from the victim's mouth; and even if our false bony skeletons fall, so that we imagine we are rid of them, we soon find them as evil spectres standing guard at our office door, crying to all comers: Who dare enter here."

It is not boasting to say that in proportion to its age, no profession today stands higher in the skill of art, in the dignity of science, and in the vantage ground of discovery, than the dental profession.

Compared with the past, it has become a profession of esthetic gentlemen. There are a few who still drink whisky, but they are ashamed of themselves. There are a a few who still chew their cud, but they are fast passing away. It is even becoming to be considered ungentlemanly to smoke tobacco in the patient's face, though eternal gum "chawing" at the chair as a prophylactic against stale tobacco stench, is almost as bad.

Society itself, has so much advanced in refinement, that to satisfy our best, and best paying customers, we must be as clean and neat in our person, habits and surroundings, and as pure and good in character, as the ladies we work for. If we have not already come up to this standard, the spirit of the age is calling us there, and giving special rewards to those who get there.

The necessity for high grade work is equally apparent. Even that it is substantial andd urable is not enough. It must be of artistic form, beautiful finish, and possess every point of skill and excellence.

And it must be put in by deft and dainty fingers. Unless we men look to our laurels, those deft and dainty fingers will be found on the pretty hands of our fair sisters, and we shall be relegated to the plow and the blacksmith shop.

Our best patients demand more than this. The dentist must be a scholar, cultured, learned and well informed in the world's affairs. Behind the instruments there must be not only the man of skill, but a well rounded man of intelligence and suavity. And what our best patrons demand, we must be; for they alone give good character and golden hue to our business.

He must also be a man of society—not a fop, a dude, a dandy, a mountebank—but a pattern of social affability and refinement, of politeness and gentility, of grace and manliness. Heretofore he has been considered too much a man "to hire," a menial, a grim, heartless personification of torture, to be avoided if possible. But now he appears with manners so gentle, with disposition so kind, and with manipulations so skillful that his work is relieved of much of its sting; and that which must be painful is ameliorated by carefulness, sympathy and medication. Then, too, if he is what he may and should be, he is more a master than a servant. And instead of receiving the wages of ordinary work, he is paid as an expert.

Time is fast coming when a man who cannot fill such a sphere will have a poor place in the profession.

Our colleges are requiring of students greater learning and manipulative skill, and more cleanliness and refinement. They are clearing their halls of vulgar loafers, and of the slaves of tobacco and beer. Can we imagine a lady student of such a character? And why should we have a lower standard for the boys than for the girls?

It may be after a long time, if we were good children, and learn better our "preliminary" lessons, that our venerable English brethren will put down their cudgel and let us land on their sacred shore—if we will agree not to take their sovereigns away from them by doing better work than they can.

Our examining boards are becoming more and more stringent in their demands, and the whole profession are reaching toward greater attainments. We are all fast clearing away the brush, setting out beautiful trees, and improving our surroundings. The public appreciate our efforts and are building for us beautiful mansions, and welcoming us as their worthy co-workers. They are beginning to recognize us, not only as a "skilled profession" and a "learned profession," but as a profession of gentlemen and social benefactors, who, as much as physicians, are spending our lives in curing and preventing the ills to which humanity is heir, and assisting to a higher civilization and the development of a nobler manhood.—*Southern Dental Journal.*

